# Cyclist Endofibrosis (Exercise-Induced Arterial Endofibrosis) Treated by Drug-Coated Balloon Angioplasty

**DOI:** 10.1155/2020/4290271

**Published:** 2020-07-07

**Authors:** Ahmed S. Zugail, Hossam I. Shaabi, Slimane Idir, Jean-Pierre Becquemin

**Affiliations:** ^1^East Paris Vascular Institute (IVPE), Paul d'Égine Private Hospital (Ramsay Santé), Champigny-sur-Marne, France; ^2^Department of Surgery, Faculty of Medicine in Rabigh, King Abdulaziz University, Jeddah, Saudi Arabia; ^3^Department of Vascular and Endoluminal Surgery, Nancy Regional and University Hospital Center (CHRU), Nancy, France; ^4^Department of Surgery, Faculty of Medicine, Jazan University, Jazan, Saudi Arabia

## Abstract

Exercise-induced arterial endofibrosis is an uncommon entity that is most frequently identified in high-performance athletes, especially cyclists. We present this disease in a male professional cyclist of 22 years of age. The course of his condition, clinical manifestations, modalities of investigation, and a nonprecedent treatment plan are demonstrated.

## 1. Introduction

Arterial diseases are rare in young people, but they could appear in the form of endofibrosis in special circumstances. Initially witnessed in highly skilled male cyclists, it has been found in many other endurance athletes of both genders. Exercise-induced arterial endofibrosis (EIAE) was first described in competitive cyclists in 1985 [1]. Sometimes, it is very challenging to diagnose this disease, and failure to do so could have grave consequences both socially and career-wise. Therefore, we believe that medical and paramedical health workers such as orthopedists, neurologists, rheumatologists, and physiotherapists should be aware of this rare condition that could imitate musculoskeletal disorders. Two distinctive characteristics remain for this ailment; it affects high-level endurance athletes, and the histopathological studies show endofibrosis [2]. To this day, the pathophysiology of this disease is unknown. We would like to add that the patient agreed to the publishing of his case details and radiological images.

## 2. Case Presentation

**Figure 1:**
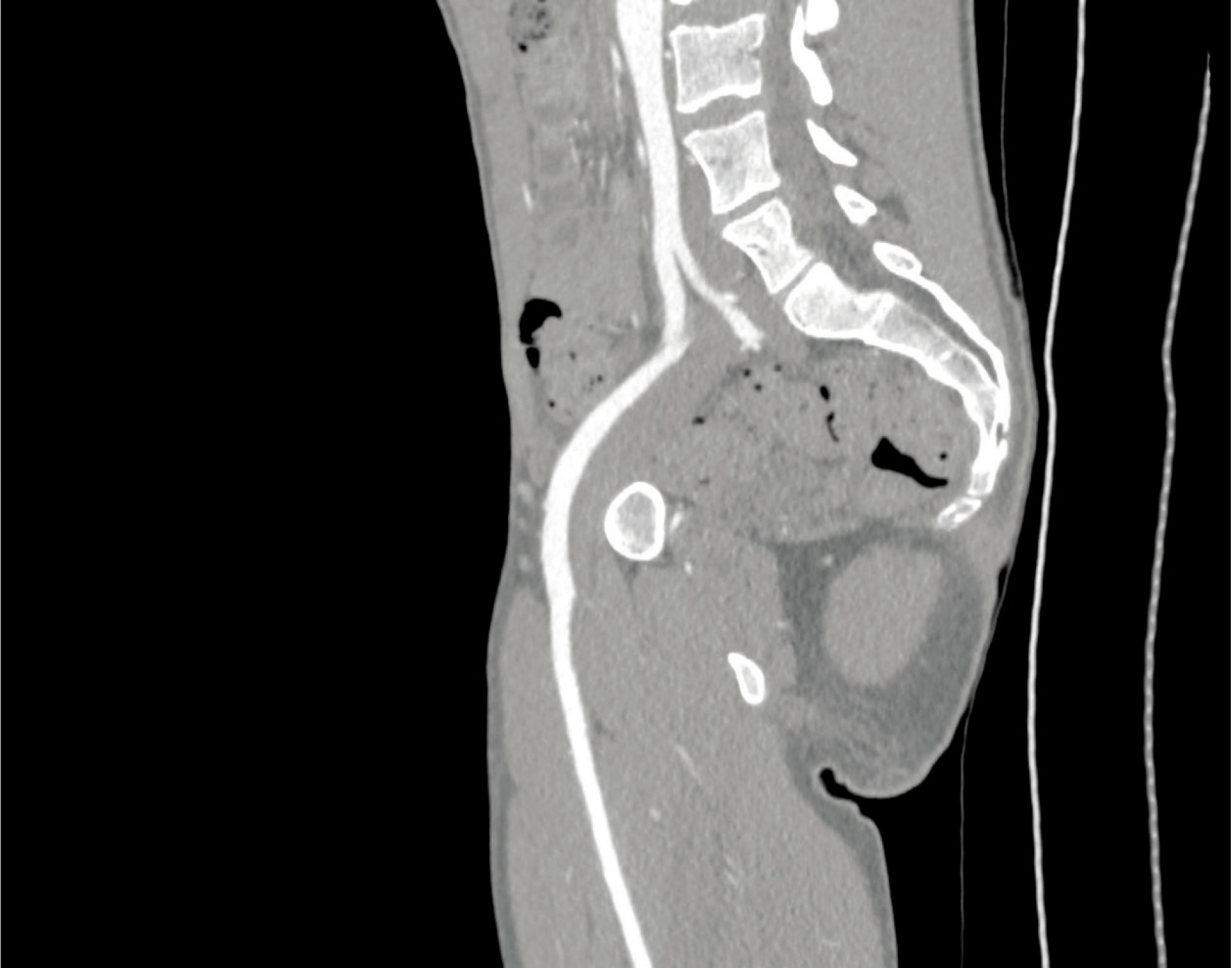
A reconstructed sagittal computed tomographic angiogram of the abdominal aorta and the left iliac arteries using the stretch reformation technique, revealing a stenosis and kink in the proximal half of the left external iliac artery (an endofibrotic plaque). Poststenotic dilation is noted. (The patient agreed to the publishing of his radiological images.)

**Figure 2:**
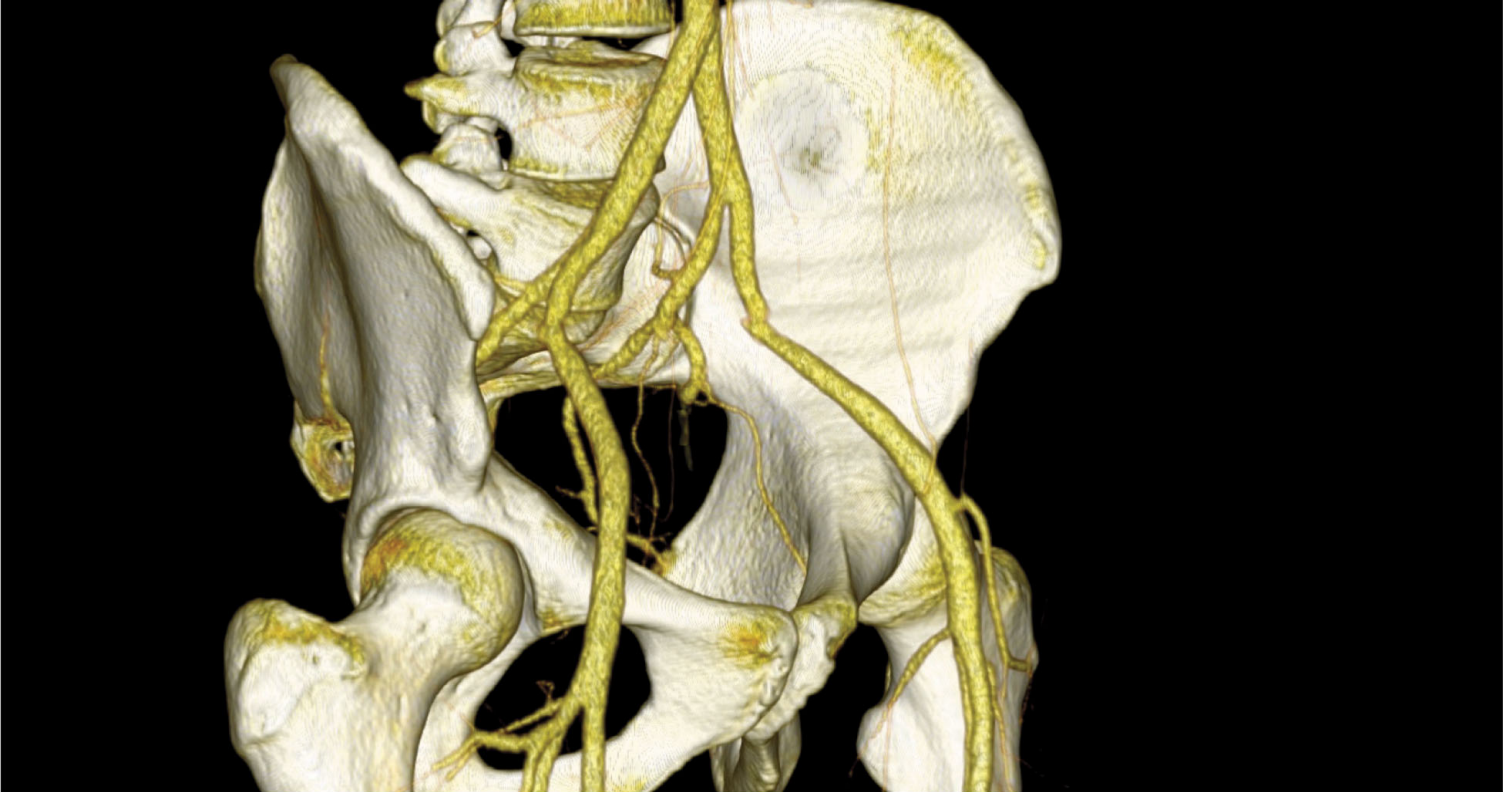
A 3-dimensional reconstruction of the patient's computed tomographic angiogram of the abdominal aorta and common iliac arteries, revealing the stenosis and kink in the proximal half of the left external iliac artery (LEA). We can observe two atypical branches of the LEA; one climbing towards the ilium and the other passing anterior to the left femoral head. Poststenotic dilation is noted. (The patient agreed to the publishing of his radiological images.)

We report a professional athletic male cyclist—more than 25,000 kilometers (km) per year—of 22 years of age with no past medical, surgical, or traumatic history who was referred by his family doctor to the vascular surgery outpatient clinic for left thigh claudication on maximal effort. The patient's symptom had already been explored with a Duplex scan and a computed tomographic angiography (CTA). The Duplex scan showed a posterior intraluminal nonatheromatous fibrous thickening at the junction of the left common and external iliac arteries of about 20 millimeters (mm) of length. At rest, no hemodynamic changes were found compared to a velocity increase at the level of the stenosis and a slight drop of the ankle-brachial index after performing 50 squats. On the CTA, an endofibrotic plaque of the left external artery (LEA) was seen Figures 1 and 2. The patient was labelled with an exercise-induced arterial endofibrosis (EIAE) or more commonly known as a *“*Cyclist Endofibrosis.” The patient was proposed four management plans given in the following order. The first was to withhold cycling which was refused by the patient. The second was surgery consisting of endofibrosectomy angioplasty with shortening of the LEA. The third was intervention by balloon angioplasty and self-expandable stent. The fourth was a balloon angioplasty using a drug-coated balloon (DCB). All the pros and cons of each treatment were discussed with the patient. He opted for the latter treatment although no scientific data supports it. The patient was admitted to the outpatient surgical department and was discharged home on the same day. After the patient's consent and under general anesthesia, an endovascular intervention was done in a hybrid operating room. An 8 mm × 60 mm LUTONIX® 035 DCB from BARD Medical, C. R. BARD, Inc. was placed over the lesion. Upon discharge, he was prescribed 75 milligrams of acetylsalicylic acid and clopidogrel once daily for one month and recommended a one-week rest before competing in a race. At the one-month clinical follow-up appointment, the patient declared no recurrence of claudication upon resuming previous cycling activity and was able to compete successfully in several races. Six months after the intervention, the patient came back with a relapse of symptoms.

## 3. Discussion

EIAE was previously known as “external iliac artery endofibrosis” as it was affected in 90% of the cases. The term was changed later since other locations were affected such as the common iliac artery, the common femoral artery, the profunda femoris, and quadricipital artery [3]. EIAE is most commonly described in cyclists; however, it has also been recognized in speed skaters, race walkers, endurance runners, triathletes, rowers, rugby players, soccer players, cross-country skiers, and body-builders [4]. EIAE is predominantly found in male endurance cyclists who are younger than 40 years of age [4, 5] and who have practiced a minimum of 45,000 to 50,000 km in more than 5 years [6]. At the onset of symptoms, these athletes will have cycled an average of 120,000 km completing between 14,500 km and 20,000 km per year [7]. Cyclists who undergo surgical treatment would have cycled a mean of 14 years and a total of 210,000 km [3, 8]. The symptoms of vascular disease in endurance athletes are seldom defined as typical claudication; they usually complain of an unexplained progressive decrease in performance and thigh discomfort in one leg or rarely both legs in 15% of cases. Sometimes, the patient describes a sensation of a swollen limb or complains of deep vague pain. Other symptoms include hardening of the thigh, cramps, and/or paralysis. Symptoms occur more commonly on the left side [9]. In 1995, Beck studied 164 cases of arterial endofibrosis and found that 50% of these patients complained of a paralyzing pain, a swollen thigh in 23%, both previous manifestations in 17%, typical claudication in 9%, and 1% had critical ischemia [10]. On the other hand, Feugier and Chevalier reported claudication of the lower limb at supramaximal exercise in 350 patients diagnosed with arterial endofibrosis [3]. All authors agree that these clinical manifestations appear during maximal effort and disappear at rest. A typical acute arterial claudication is rarely found and usually suggests a complication such as an occlusion [11] or a dissection [12]. Complications of endofibrosis include thrombosis, dissection, and secondary atheroma [3]. Direct trauma or familial history of vascular disease must be explored to exclude direct arterial damage or early atherosclerosis. Clinical examination is usually normal, but patients could present with an arterial systolic bruit at the groin at or with the thigh flexed. This sign is commonly found in highly trained athletes due to low resting heart rate and could be found in normal subjects, thus, not specific for endofibrosis. Differential diagnosis of EIAE includes atherosclerosis of familial origin, any musculotendinous lesion that can mimic claudication, fibromuscular dysplasia, compartment syndrome affecting the thigh, adductor canal syndrome, kinking of the femoral or iliac arteries, atypical sciatica with truncated symptoms, abnormal muscle metabolism that could be responsible for cramps during exercise, and femoral stress fractures. Different factors have been suggested that could explain the pathophysiology of endofibrosis; the aerodynamic posture and repetitive limb flexion during exercise may cause repeated stretching and lengthening of the artery that could lead to arterial kinking, and the presence of atypical arterial branches to the hypertrophied psoas muscle may displace, limit the mobility, or produce kinking of the artery and/or hereditary and metabolic factors [3, 4, 13]. The presence of a large sciatic artery acting like a pin around the affected artery has also been proposed [14], but this does not explain cases of femoral or quadricipital artery involvement. The increased blood flow and shear forces may cause stress injuries to the arterial wall with intense exercises. Contrariwise, these minor arterial lesions can lead to a significant flow impairment upon heavy load exercises where high blood flow is demanded due to an inverse relationship between the pressure drop and both the percentage of the stenosis and blood flow through these lesions [15]. Moreover, the significant increase in homocysteine in the blood and urine after methionine loading could suggest a metabolic predisposition as a minor dysfunction of the methionine cycle [3]. This abnormality increases the exercise diastolic blood pressure response and causes endothelial dysfunction [16]. Histologically, the lesion consists of an asymmetric intimal thickening with normal-appearing endothelium. The subendothelial layer shows coarse fibrous tissue with few cells that are mostly myofibroblasts in a milieu rich in collagen but poor in elastic fibers. The richly cellular intima with myofibroblasts suggests medialization. A neointimal elastic membrane in proximity to the arterial lumen is shown occasionally. The internal elastic membrane, media, and adventitia are usually normal, but the elastic membrane could be duplicated [14]. These histopathological features cause stenosis of the artery thus restricting the blood flow to muscles and produce the symptoms during maximal effort [2]. Numerous methods are used to diagnose EIAE, exercise testing with the Doppler velocimetry or ultrasound imaging, which will confirm the acceleration of blood flow with low frequencies on the spectral analysis due to turbulence [17]. Typical ultrasound imaging features include parietal thickening, enhanced echogenicity of the arterial wall, straightness of the abnormal arterial segment, and mild narrowing of the arterial diameter of the proximal or medial segment by using the B-mode that has 78% sensitivity and an 80% specificity for EIAE diagnosis [18]. In most cases, the Doppler velocimetry or ultrasound imaging at rest shows normal profiles. Furthermore, postexercise ankle-brachial systolic pressure index (ABI) is an important diagnostic tool; at rest ABI is normal. ABI measurements taken in the supine position that are less than 0.5 after completing a one-minute exercise can identify 80% of cases with 100% specificity for EIAE [4]. Little is known about computerized tomographic scans and magnetic resonance imaging of EIAE; as a result, most patients have been studied with arteriography. With the widespread of CTA and magnetic resonance angiography (MRA), arteriography with the Seldinger technique preoperatively is becoming obsolete. Sensitivity and specificity of CTA in detecting 50% aortoiliac stenosis, for example, are 96% (95% CI: 91–99) and 98% (95% CI: 95–99) [19]. The role of MRA is limited to those patients without identified endovascular lesions and whose symptoms may be due to arterial kinking [20]. Angioscopy (endovascular ultrasound) has been performed in a large number of patients showing a large eccentric white regular plaque and defining its extensions [10]. This modality needs to be broadly studied since it is certainly a promising technique although it is rarely used nowadays. The two most commonly used methods to repair EIAE are endofibrosectomy with patch angioplasty or interposition grafting with recalibrated saphenous vein grafts providing long-term satisfactory results [3]. Polytetrafluoroethylene (PTFE) prosthetic grafts should not be used in treating EIAE because almost all prostheses have occluded in the experience of Chevalier et al. and Beck [10, 14]. Percutaneous balloon angioplasty may be used when temporary relief is needed, but patients generally have recurring symptoms within 8 weeks of treatment [21]. We believe that this is the first case of EIAE treated primarily with a drug-coated balloon (DCB) angioplasty after reviewing both the English and the French literature. We believe that this method should be suggested for these patients because it is minimally invasive, relatively safe, and easily reproducible and has in the current case an excellent immediate outcome, but further studies are indispensable to confirm its long-term efficacy. In conclusion, EIAE is an uncommon ailment and is typically diagnosed in high-performance athletes, especially cyclists. The condition should be considered early in athletes who experience reproducible symptoms with exercise. Failure to diagnose and treat EIAE in young athletes has both career and social implications; a high index of suspicion for this condition is important. Ultrasound, the Doppler, and ABI studies should always be performed at rest and after exertion. CTA in appropriate patients, MRA, should be done systematically. No guidelines are available for the management of EIAE; thus, all treatment modalities should be discussed carefully with the patient including DCB angioplasty.
